# Supplementary material for: Electro-optic imaging enables efficient wide-field fluorescence lifetime microscopy
Source: Nat Commun. 2019 Oct 8;10:4561. doi: 10.1038/s41467-019-12535-5 (PMC6783475; doi:10.1038/s41467-019-12535-5)
Supplement: Supplementary file 1 — Supplementary Information [file 41467_2019_12535_MOESM1_ESM.pdf]

Supplementary Information

Electro-optic imaging enables efficient wide-field fluorescence lifetime microscopy

Bowman *et al.*

Supplementary Note 1: Calculating isogyre patterns

Isogyre or conscopic interference patterns are calculated following West [22]. For a general electro-optic crystal, the indices of refraction are modified by electric field $\vec{E}$ as $\Delta(1/n^2)_i = r_{ij}E_j$ where r_{ij} are the elements of the contracted electro-optic tensor. The general index ellipsoid is written as

$$\left(\frac{1}{n_{x_1}^2} + r_{1j}E_j\right)x_1^2 + \left(\frac{1}{n_{x_2}^2} + r_{2j}E_j\right)x_2^2 + \left(\frac{1}{n_{x_3}^2} + r_{3j}E_j\right)x_3^2 \\ + 2x_2x_3r_{4j}E_j + 2x_3x_1r_{5j}E_j + 2x_1x_2r_{6j}E_j = 1. \quad (1)$$

For KD*P ($n_\mathrm{o} = 1.5079; n_\mathrm{e} = 1.4683; r_{63} = 26.4$ pm/V) in longitudinal configuration ($\vec{E}$ and optical axis along x_3), only r_{63} contributes. We can then consider the effect of off-axis rays by applying rotations in x_1 and x_2 such that $\vec{\mathbf{y}} = R_{x_1}(\beta)R_{x_2}(\alpha)\vec{\mathbf{x}}$. Setting $y_3 = 0$, the transformed index ellipsoid becomes

$$A_1y_1^2 + A_2y_1y_2 + A_3y_2^2 = 1 \quad (2)$$

with

$$\begin{aligned} A_1 &= \frac{1}{n_0^2}\cos\beta^2 + \frac{1}{n_e^2}\sin\beta^2 \\ A_2 &= \left(\frac{1}{n_0^2} - \frac{1}{n_e^2}\right)\sin 2\beta\sin\alpha + 2r_{63}E_3\cos\beta\cos\alpha \\ A_3 &= \frac{1}{n_0^2}(\sin^2\beta\sin^2\alpha + \cos^2\alpha) + \frac{1}{n_e^2}\cos^2\beta\sin^2\alpha + r_{63}E_3\sin\beta\sin 2\alpha. \end{aligned} \quad (3)$$

The index ellipsoid is then transformed into the basis of the principal axes z_i to become $C_1z_1^2 + C_2z_2^2 = 1$. This new ellipsoid is rotated by angle $\rho = \frac{1}{2}\cot^{-1}\left(\frac{A_3-A_1}{-A_2}\right)$ relative to the on-axis index ellipsoid, giving [22]

$$C_1 = A_1\cos^2\rho + A_2\cos\rho\sin\rho + A_3\sin^2\rho \quad (4)$$

$$C_2 = A_1\sin^2\rho - A_2\cos\rho\sin\rho + A_3\cos^2\rho. \quad (5)$$

The semi-major and semi-minor axes of the transformed index ellipsoid give the new indices of refraction $n_{z_i} = 1/\sqrt{C_i}$. The phase shift for the off-axis ray defined by transformation angles β and α is then $\delta = \frac{2\pi d}{\lambda}(n_{z_1} - n_{z_2})$. To evaluate the conscopic interference pattern, this phase shift is used as input to the Mueller matrix for a linear retarder $\mathcal{R}(\rho,\delta)$ [21] and the intensity for off-axis rays through crossed polarizers is found using Stokes vectors and the Mueller matrix formalism as $S_\mathrm{out} = \mathcal{P}\mathcal{R}(\rho,\delta)S_\mathrm{in}$ where $\mathcal{P}$ is a polarization analyzer perpendicular to S_{in}. To model the effect of static compensation with MgF_2 ($n_\mathrm{o} = 1.3786; n_\mathrm{e} = 1.3904$), it's off-axis Mueller matrix was calculated (identical process, except no applied field) and included as $S_\mathrm{out} = \mathcal{P}\mathcal{R}_{\mathrm{MgF}_2}(\rho,\delta)\mathcal{R}(\rho,\delta)S_\mathrm{in}$. Results for thick, thin, and MgF_2 compensated KD*P Pockels cells are shown in Supplementary Figure 1.

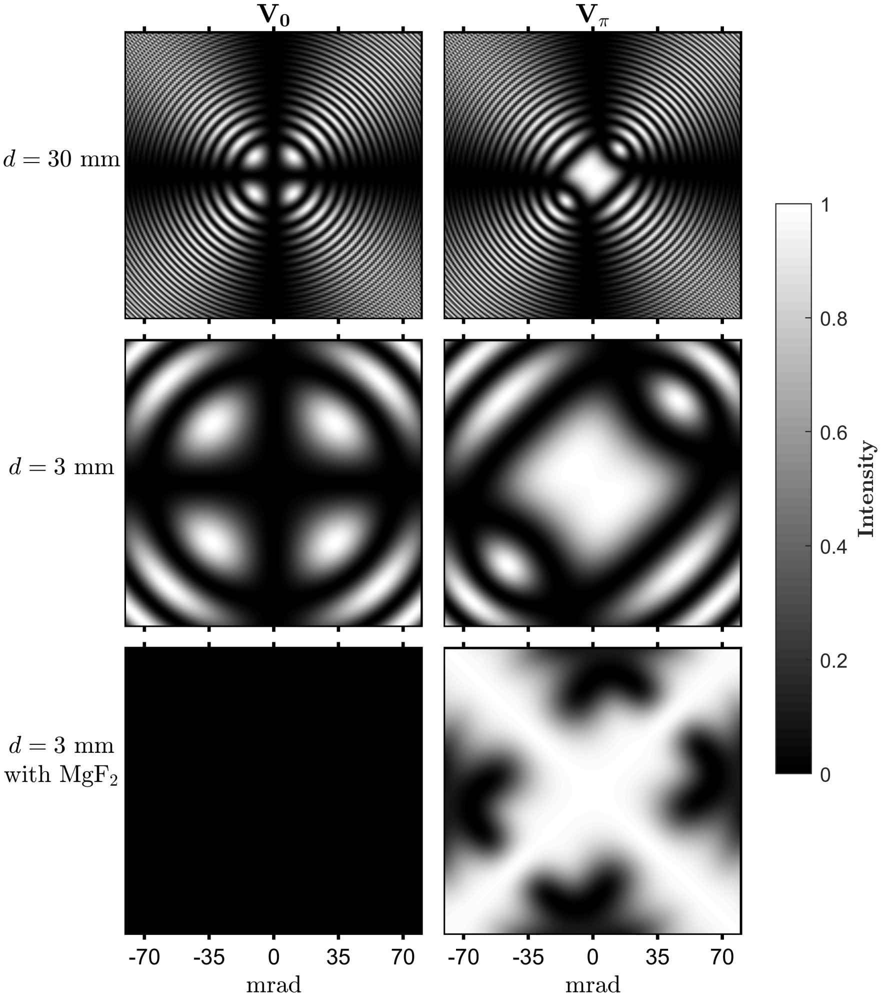

Supplementary Figure 1: Longitudinal KD*P isogyre patterns and off-axis compensation Isogyre patterns are shown for longitudinal KD*P Pockels cells at V_0 and V_π (transmission through crossed polarizers). The central region of the pattern corresponds to the good NA of the cell where gating efficiency of 1 is obtained. KD*P becomes biaxial under electric field which breaks the pattern's symmetry in the right column. In the second row, we see that thinning the crystal to $d = 3$ mm increases the half acceptance angle α to ~ 20 mrad. Addition of a matched MgF_2 crystal of thickness 10.6 mm in the third row gives complete cancellation of off-axis phase shift at V_0 and partial improvement at V_π. Thin and compensated cells can thus provide further improvements in image field-of-view or NA depending on the PC imaging modality. These improvements could be particularly important in intracavity gating of tilted mirror cavities for ultrafast imaging.

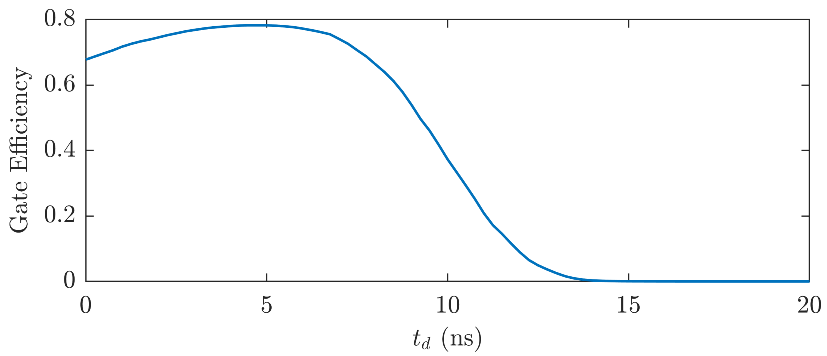

Supplementary Figure 2: Gating function for 3 mm KD*P Pockels cell Instrument response function (IRF) for the thin crystal demonstration in Figure 4.

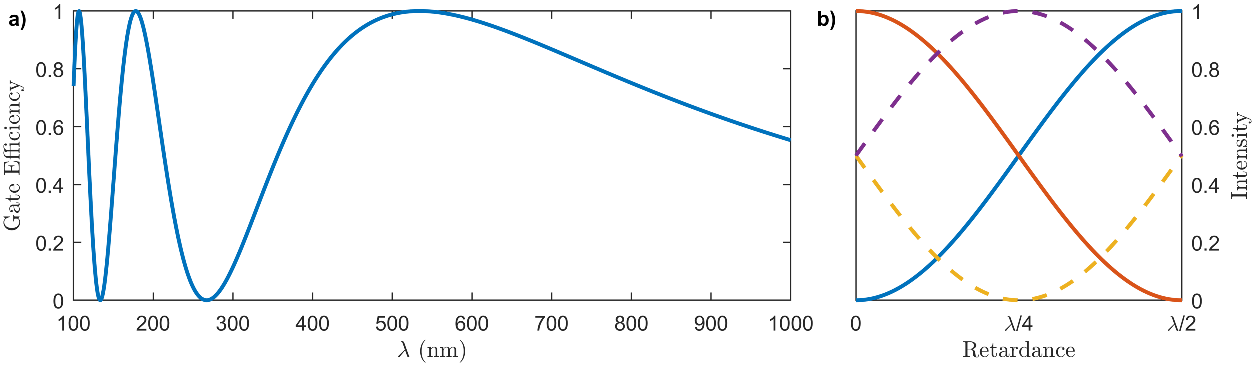

Supplementary Figure 3: Gating as a function of wavelength and retardance (a) Spectral dependence of PC gating efficiency with V_π set for 532 nm. High efficiency gating is shown over a ∼100 nm wavelength range compatible with fluorescence emission spectra.(b) Intensity of output image channels as a function of PC retardance. In the case shown in Figure 1(a), crossed polarizers result in pairs of output frames who's intensities follow the two solid lines. If a quarter waveplate is inserted at one of the outputs of the PC before the second PBS, then two of the channels can instead follow the dashed lines. This operation mode would be advantageous for a ramped V(t) to streak polarizations and temporally localize bursts of photons on sub-framerate timescales. Without the QWP, the temporal sensitivity is not optimized over the entire range of retardance. Further, we note that the QWP outputs are wavelength sensitive near 532 nm, whereas the gating efficiency in (a) is wavelength insensitive. This could potentially be exploited for multi-dimensional measurements of spectrum and lifetime.

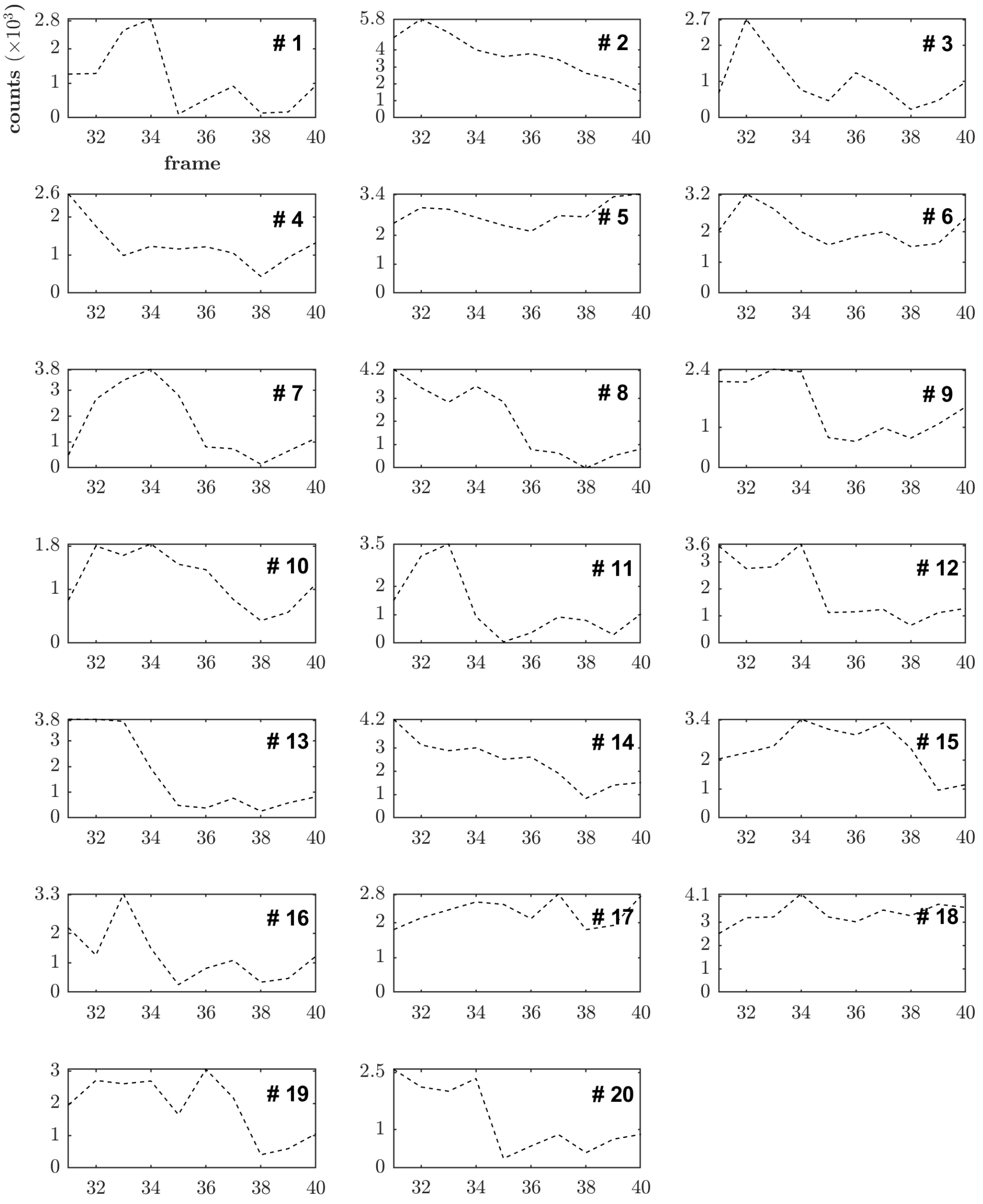

Supplementary Figure 4: Single-molecule intensity traces and bleaching Time traces of intensity are shown for all the diffraction-limited emitters numbered in Figure 3(a and b). Each frame corresponds to a 5 second exposure at 15 kHz repetition rate. While this timescale is far to long to see molecule blinking as typically observed, we are able to observe step-like bleaching events in many of these emitters (i.e.#'s 1,3,7,8,9,11,12,13,16,19, and 20) and even temporary brightening or re-activation from the dark state (#'s 1,3,7,11, and 16). Certainly some of these spots (like the brightest,# 2) likely contain multiple emitters, but we find the majority show signals and behavior consistent with single molecules. Supplementary Figure 4 shows sequential image frames with examples of the same dynamics.

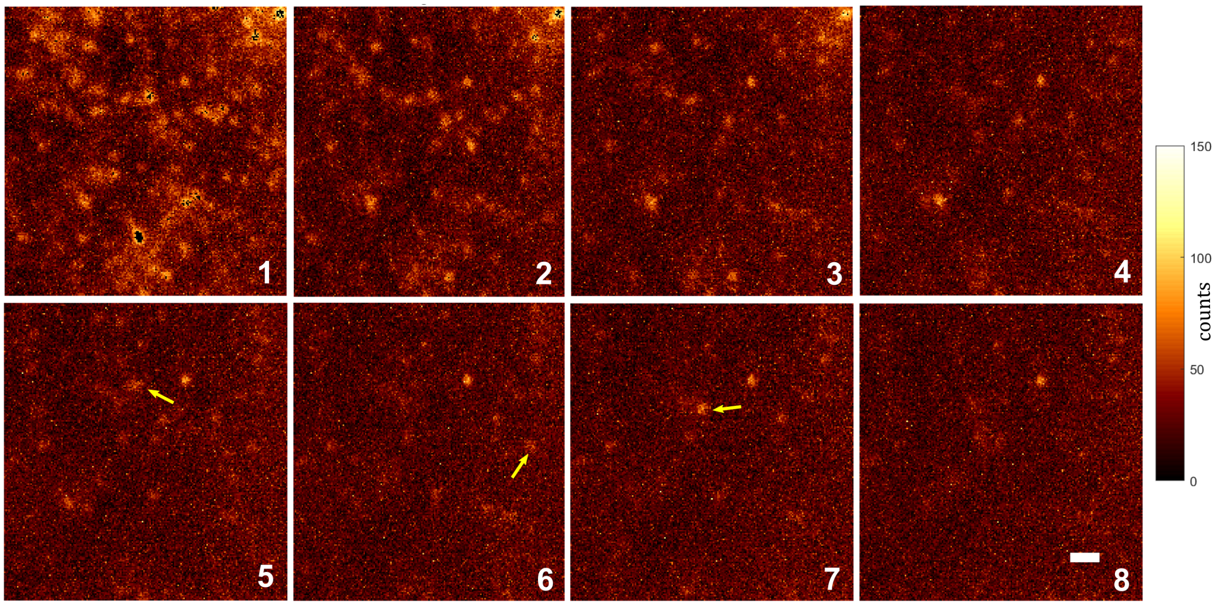

Supplementary Figure 5: Image sequence of molecule bleaching A sequence of eight frames (5 s exposure each) demonstrates photobleaching and dynamics of Alexa 532 molecules. Molecules can undergo single-step bleaching between sequential frames. Here the field is bleached down as frames progress from 1 to 8. A few molecules (indicated with arrows) re-activate from the bleached state (scalebar 1 μm).

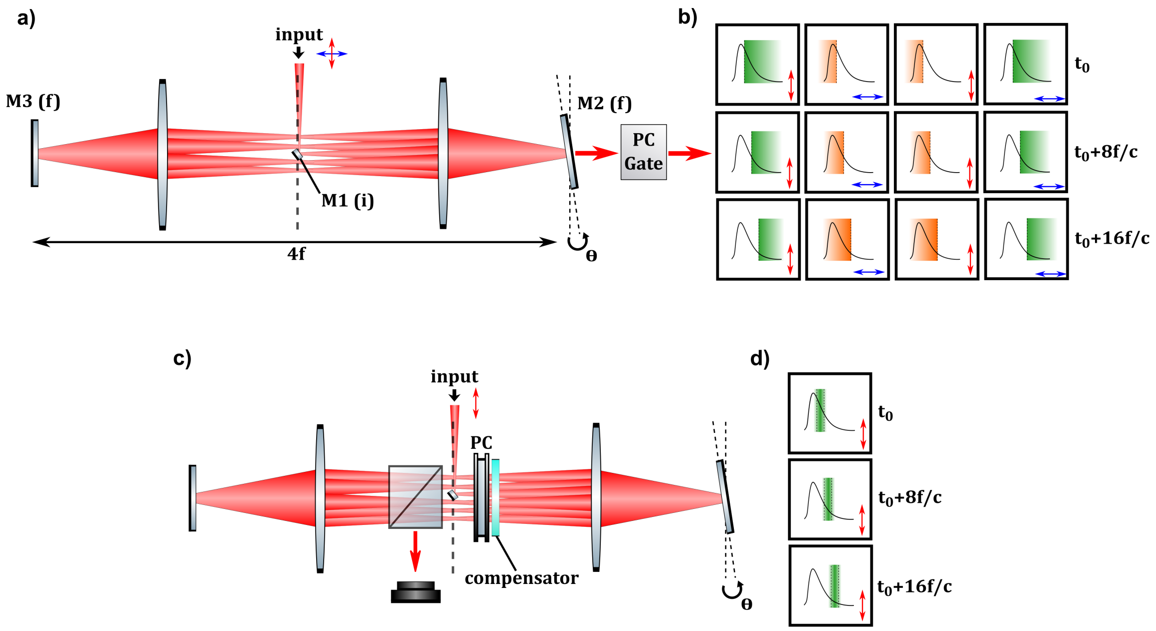

Supplementary Figure 6: Schematics for internally and externally gated tilted mirror re-imaging cavities (a) Reproduced schematic of externally gated tilted mirror re-imaging cavity from Figure 4. Note that while we use a PC external gate, any gate can work if efficiency is not a concern (standard gated optical intensifiers for example). (b) Using the PC gate, four image frames (columns) are formed from each cavity output as shown in Figure 1. Shaded regions show collected intensity in each image frame for a step function gate and a fluorescence decay. Note that for each row the gate occurs at a different time relative to the decay signal. This is due to the addition of round trip delays. (c) Internally gated re-imaging cavity proposed in the text. A birefringent compensating crystal is illustrated along with the PC near the central image plane. This scheme removes passive outcoupling through a transmissive mirror and instead gates all light out of the cavity with the PC and a PBS. (d) Shaded regions of the fluorescence decay show distinct temporal bins captured in each cavity output. As drawn, the temporal bins would have width $1.5\,t_{\rm rt}$ and outcoupling would be accomplished with a $V_{\pi/2}$ pulse on the PC. If the PC is moved near a mirror, then bins of width $t_{\rm rt}$ without overlap are obtained. In both cases the PC is double-passed before light is outcoupled. Here, the tilted mirror cavity acts as an n-frame ultrafast camera with zero deadtime between frames. The risetime of the PC leads to some overlap between the temporal bins in the output images. To collect all emitted photons, polarizations would be split and rotated before the cavity. Thin, compensated PC crystals are ideal for this application.
